# Improving communication for learning with students: expectations, feedback and feedforward

**DOI:** 10.15694/mep.2019.000014.1

**Published:** 2019-01-15

**Authors:** Tim Ley, Jolanta Kisielewska, Tracey Collett, Steven Burr

**Affiliations:** 1 Peninsula Medical School

**Keywords:** feedback, feed up, feed in, feedforward, managing expectations, formative assessment, communication

## Abstract

This article was migrated. The article was marked as recommended.

The aim of this paper is to draw together numerous strands from within the literature and our own practice to provide advice for improving communication about learning with students in undergraduate medical education. There is an assumption within higher education that assessment drives learning and, as such, assessment forms the focal points for communication between teachers and students. However, a broader approach is required to avoid misunderstandings and maximise the successful engagement with learning of everyone involved. It is important to plan a clear communication strategy that incorporates and enables identification with the unique values of the particular school. Where communication about learning is overtly discussed there are three main areas to consider: (1) management of expectations (sometimes referred to as feed up or feed in) that needs to include not only the use of authentic formative assessments, but also the viewpoints of both teachers and students. (2) Feedback and (3) Feedforward, both need to be considered from the perspectives of student and teacher. All communication needs to be inclusive, it’s structures must provide scaffolding for respectful exchanges of information, and this will have clear practical consequences for the activities within the school.

## Introduction

Education can be conceptualised as a flow of information. In a traditional information transfer model, the information is passed from teacher to student who then demonstrates they have learned what is required by passing the information back to teachers in assessments ( National Research Council, 2001). More modern conceptions of education differ from this model in several important respects. One difference is that education has come to be seen as an interaction between teacher and student, or even a partnership ( Bryson, 2016). In order for this relationship to be effective, the views and wishes of students have to be considered. The transfer of information is thus bi-directional (teacher to student and student to teacher) at many stages within the process of education. Various types of information conveyed from teachers to students have parallels in the information conveyed from students to teachers.

The different forms of information that are provided to students will now be considered. The assessments within a programme have the purposes of determining the level of student attainment and providing a goal for student learning activities. Assessments can also be conceived as focal points of communication between teachers and students. There should be three main strands of formative information in any curriculum: (1) Statements of expectations, sometimes now referred to as feed in or feed up ( Fisher & Frey, 2009; where the students are fed up), is the provision of information on what is required for success. The term ‘feed up’ has also been used to denote feedback, intended for use after studies have been completed ( Evans, 2013) and we will avoid further use of this term ourselves. (2) Staff feedback is the provision of information by teachers on performance, saying what the student has achieved and what can be improved, relative to what was required; and (3) Staff feedforward is the provision of information on performance intended to develop ability for future requirements. To guide students to their best possible performance requires effective combination of clearly expressed expectations, feedback, and feedforward.

## Take a strategic approach to the communication of information about learning within the programme as a whole

Key to providing good quality education is effective communication to students about their learning in terms of what to learn, how to learn, how well they have learned, and how to move learning forward. The overall flow of information within a whole programme can be planned; this is in marked contrast to the current norm where elements of the course are modularised into discrete units with separate informational processes. Thus, it is important to write a learning communication strategy, to ensure that all information about student performance is visible as a core thread throughout the curriculum, transparently mapped to the learning outcomes. This can be achieved by planning much of the exchange of information, including module feedback, both formative and summative, so that it informs subsequent modules. This would prioritise the longitudinal development of students, mapping, monitoring and supporting their growth towards the target learning outcomes in knowledge, clinical skills, and professionalism. The best time to plan information flow in a programme would be at its revalidation, but post hoc reflection and development is also useful.

We advocate the production of a programme scheme regarding feedback, to show how feedback on work can inform other modules. Map how each module informs those that follow it in terms of both the timing and nature of the feedback that is produced. In order to achieve this, it is necessary for teachers to be clear about the transferable skills associated with their topics ( Watson & Burr, 2018). It is very easy for teachers to be conscious only of the specifics of their discipline and be relatively unaware of the general lessons that they teach. Only when teachers appreciate the generalisability of the material they teach, can they provide the most useful feedforward and equip students for future assessments. All teachers need to be aware of the spiralling in the curriculum where topics are revisited with increasing levels of sophistication.

In essence, what is needed is a streamlined framework for exchange of learning information. This can be supported when teachers deliver their material with a positive attitude and demeanour ( Naftulin et al., 1973; Merritt, 2008). Engagement with both the receipt and provision of feedback develops student ability and confidence. For example, it may improve a student’s understanding of learning outcomes from the perspective of others. This process can be supported by mutual agreement of points for interaction between students and teachers. Ground rules for these interactions should include reciprocity of engagement, and the acceptance of both expert academic judgement and the regulations of the institution.

## Embody the school’s aspirations within the communication strategy

A communications strategy can be an embodiment of the school’s aspirations for its relationship with students. The NUS (2015) document, the “Assessment and feedback benchmarking tool”, is a document that powerfully expresses the NUS’s aspirations regarding the place of students as partners in the education process. This vision of partnership may, or may not, be fully appropriate within the context of education for healthcare professionals. Students are being prepared for future professional roles within the health service and the choice of the material they learn and their assessment are strongly influenced by validating bodies and the health service. Whatever vision a school has of its relationship with its students, this should be reflected in its strategy for communication. The acts of the school in planning the flow of information in a programme will speak as clearly as any carefully written vision statement. The places that statements of expectation, feedback, and feedforward, take within the programme can make a difference to the culture of the school as a whole.

## Optimise communication of the expectations that teachers have of students

The ethos of the programme needs to be clear to students before they decide to join. Once enrolled it is important to explain the rationale behind both the overall and detailed programme requirements in order to manage student expectations and perceptions of communication during their studies. It is necessary to be explicit about the constructive alignment of both programme goals and distinctiveness, and also the obligations of both students and teachers. Everyone should know what success looks like before they start ( Hattie, 2015) and be regularly reminded. There should be an understanding that what constitutes a successful student performance profile can vary and evolve. The communication of expectations is therefore much more than the provision of documentation in advance, for example on assessment tasks, marking criteria, and regulations, ( Nicol and Macfarlane-Dick 2006). Such communication requires a thorough understanding of purpose and means within the programme. This is facilitated through all points of interaction between a student and the experiences their school provides.

However, it is easy to overwhelm by providing too much regulatory information. There is a modern bureaucratic tendency, to burden everyone with too many long documents in an attempt to permit or prevent various actions and mitigate against complaints and appeals on the basis that accountability wasn’t clear in advance. Too often no-one has time to read these documents until after they have a problem, and the system becomes undermined by a sense of taking sides and legalistic game playing. Therefore, where possible, simplify processes and prioritise an active dialogue of equally shared responsibilities throughout ( Laurillard, 2002). The provision of opportunity to demonstrate ability at repeated and escalating levels of challenge coupled with feedback, that clearly link student motivation to programme goals, can drive a more authentic approach to learning ( Lombardi, 2007).

## Optimise understanding of student expectations

In order to guide students effectively, it is important to take account of what individuals want to know and what they don’t want to know, alongside how they define success, and how this is changing. All students want to pass, and want fair (accurate and non-discriminatory) assessment of what they have learnt. There is a fear of failure and the emotional impact of ‘taking a hit’. However, failing to fail ( Yepes-Rios et al, 2016) robs students of the opportunity to develop capacity to deal with the anxiety of failure. It is similarly important to encourage students to own up to not knowing. Rather than to complain about the curriculum or its delivery. Students need to be aware of, and reflect on, their own learning processes and thus to develop skills to solve problems ( Bransford et al, 2000). When a teacher admits that they don’t know, this provides a lesson for students and creates an environment of trust for shared learning. To reward co-operative practices can itself lead to competition and conflict, by identifying who helps others most. If performance is ranked it is deemed higher stakes, and creates a stronger motivation to criticise the fairness of processes. It is thus important to check with students that the intention behind communication processes aligns with the student experience. If the student prioritises a performance-centred approach, they will strive to avoid criticism and maximise their score; whereas if taking a learning-centred approach they may seek out new challenges and, whilst scoring lower, develop their competence further ( Dweck, 1986). A learning-centred approach can be encouraged by formative assessment. Competitive practices such as ranking, whilst authentic to the workplace, can create an unwelcome culture amongst students enlarging the informal (hidden) curriculum ( Lempp & Seale, 2004). Mechanisms are needed to minimise any negative impact of the hidden curriculum and ensure the fair distribution of information to all. Self-directed peer group learning activities can produce rumour and myths about how to achieve success, which need to be dispelled. Equally, these activities can also produce alternative means to achieve improvement that teachers are unaware of, and these need to be shared, to ensure all students are aware and have an equal opportunity to develop.

Life as a medical student is not always as they expect it to be and there needs to be an understanding that as learners they will have challenges during their education. The actions of teachers therefore will not, and should not, be wholly directed by the wishes of the students as they progress through the programme (Furedi, 2012). Negotiating the balance between teacher and student expectations regarding issues like this can be difficult. A very rigid approach from teachers can lead to resentment and potentially poor engagement from students. It is important that students feel that their perspective is heard and considered. When it is not appropriate to accept the majority student perspective, it is doubly important to make clear why that decision has been made and the process by which that decision will continue to be reviewed, if that is the case. Where student expectations are a driver for policy, it is important to make this fact clear to students, in order to foster good staff-student relations. Negotiations regarding topics such as this can have a great importance in developing the relationships between teachers and students and can also be a vehicle to form the students’ expectations of the future.

## Ensure a wholly formative opportunity precedes every type of summative assessment to minimise misconceived expectations

The emphasis on formative and summative feedback should be equal, as should be their quality. Feedback on formative performance should thus mirror, and explicitly inform, each form of summative assessment. For example, a past and complete summative test can be delivered under full examination conditions as a prelude to the first summative test. If necessary, in order to provide an adequate time to receive feedback and remediate, this formative test can occur even before all of the syllabus has been covered. Such a test is then not only an authentic practice at the format and conditions of delivery, but also provides fair notice of the content breadth and depth that can be expected in the summative test; a valuable ‘wake-up call’ for some students. Performance can be enhanced by supporting the strategies adopted by the student when preparing for the test and during taking the test. Thus, ‘assessment literacy’ is improved ( Price et al., 2012). An example is to receive formative feedback regarding professionalism before a summative assessment. This gives the student opportunity to improve and respond to feedback. Assessments in medical education comprise writing reflective pieces, the development of professional and clinical skills as well as tests of medical knowledge. The broad spectrum of different types of assessment could confuse students and therefore it is fair to explain to students what educators want to achieve and how medical students could use particular types of assessment in future career development.The emphasis on formative and summative feedback should be equal, as should be their quality. Feedback on formative performance should thus mirror, and explicitly inform, each form of summative assessment. For example, a past and complete summative test can be delivered under full examination conditions as a prelude to the first summative test. If necessary, in order to provide an adequate time to receive feedback and remediate, this formative test can occur even before all of the syllabus has been covered. Such a test is then not only an authentic practice at the format and conditions of delivery, but also provides fair notice of the content breadth and depth that can be expected in the summative test; a valuable ‘wake-up call’ for some students. Performance can be enhanced by supporting the strategies adopted by the student when preparing for the test and during taking the test. Thus, ‘assessment literacy’ is improved ( Price et al., 2012). An example is to receive formative feedback regarding professionalism before a summative assessment. This gives the student opportunity to improve and respond to feedback. Assessments in medical education comprise writing reflective pieces, the development of professional and clinical skills as well as tests of medical knowledge. The broad spectrum of different types of assessment could confuse students and therefore it is fair to explain to students what educators want to achieve and how medical students could use particular types of assessment in future career development.

## Optimise feedback to students

A great deal has been written about the provision of feedback to students. Feedback should, but often isn’t, expressed in language that is meaningful to the student ( Orsmond et al, 2011). In doing so we need to take into account cultural and other differences among our students so that the feedback is appropriate to the individual that receives it. Feedback can be written in such a way as to encourage self-sufficiency rather than dependency in students; for example, by using questions to the student, rather than statements of fact ( Carless et al, 2011). If the feedback to the student is not congruent with the mark that accompanies it this can cause a sense of injustice in the student ( Ferguson, 2011). Thus, efforts to provide ‘tactful’ feedback may misfire, if they result in an inconsistent message to the student. While detailed feedback is sometimes seen as the ideal, too much detail can lead to a student feeling overwhelmed and unable to see where the focus of future effort should lie ( Ferguson, 2011). Optimising feedback requires teachers to: (1) Provide information on assessment and feedback individually, and for the cohort, compared to other cohorts at the same stage and all other stages to compare growth trajectories. (2) Provide timely help as close to the event as possible to maximise recall and impact ( Race, 2007). (3) Ensure criticism is constructive and balanced by praise where there any grounds to provide it. ( Hattie and Timperley, 2007). (4) Be direct, acknowledge what went well, and mutually explore what needs to improve and how this could be achieved; convey recognition and recommendations based on evidence using specific observed examples. And, (5) Where possible always finish by confirming understanding, both theirs and yours ( Nicol, 2010).

## Optimise feedback to teachers

Teachers learn from the students. Frequent review provides multiple opportunities to identify difficulties and remediate ( Ricketts & Bligh, 2011), not only in student performance, but also in the performance of the curriculum. Student feedback can inform curriculum planning and faculty training, and enable teachers to evaluate the effectiveness of their teaching, and improve opportunities for all. Surveys need to be used sparingly, with clearly articulated purposes and potential outcomes, to avoid fatigue ( Porter et al., 2004). In particular, if Likert scales provide the opportunity to give moderate, possibly undecided, responses; with the majority of responses in the middle, a dissatisfied minority can skew the overall perception that is apparent from a group mean. This difficulty can be reduced if results are summarised with a median that reduces the effect of such outliers. It is also constructive for a threshold for action to be articulated to all in advance. When teachers seek feedback, they should reaffirm the distinction between learning and performance, and the rationale for current practice, for example, of not normally coaching students for exams. It can be useful when managing teacher wellbeing to categorise feedback into actionable, non-actionable, and ‘faint praise’, and filter out comments that are not constructive. Get students to feedback their understanding of their results. It is helpful to know how failures are perceived, because how a student perceives failure will influence his or her future behaviour. It is therefore worth determining if the failure is seen to be due to failure to teach, failure to provide appropriate assessment processes, insufficient student aptitude, student application, or extenuating circumstances.

## Optimise feedforward to students

It is frustrating for both students and teachers when feedback on student work is not, or cannot be, acted upon, especially as it results from so much effort, from both students and teachers ( Sadler, 2010). This difficulty may be minimised when the aim is to establish a transparent integrated feedforward narrative across a whole programme. This can be achieved by reviewing all points of feedback to students throughout the programme and integrating these into a narrative to maximise the utility of the information provided. If it were clear to both markers and students how and when feedforward could be acted on within the programme, this would provide a purposive focus. Thus, it is necessary to ensure that the role of information in guiding future work is explicit to both students and teachers. Therefore, the use of formative and summative information, the intention behind it, and how that intention will be achieved, should all be covered within module documentation and assessment guidelines. The overall aim is to encourage self-regulated learning and to prevent ‘learned dependence’ ( Yorke, 2003).

## Optimise feedforward to teachers

Student ideas for changes to a programme cannot mandate its future direction, but they can form an excellent starting point for teacher reflection. The behaviour of both teachers and students are strongly affected by their separate cultures and it is easy for both parties to assume that the perspective of the other is based on ignorance of the facts. The mechanisms embedded within a programme should allow the collation of student views about blocks of teaching and their place within the programme. Student comments to individual teachers, about blocks of teaching and about the programme as a whole may carry a common message. If the different elements are responded to piecemeal, any consensus among student views may be lost to teachers. Places where student ideas can be discussed include, of course, staff meetings with student representatives. The dialogue that can occur here can either instigate change or foster mutual understanding. For example, student suggestions regarding timetabling can show that current arrangements, although flawed, are the best compromise given the various constraints. It is only by talking through the issues that the rationale for current arrangements can be clear to students. On other occasions, student perceptions may lead to immediate change, for example, a student complaint that two very challenging sessions were back to back in the timetable was enlightening for teachers who previously had not recognised that one of the sessions could be perceived as particularly difficult. Student comment can also reveal areas for potential improvement in teacher performance and this can influence plans for teacher training, either on an individual or team basis.

## Ensure the formats of all communications are inclusive, or tailored, to maximise understanding

We believe that that the stigma of disability for medical students and trainees has reduced somewhat, and this may account for the increase we are observing in the number of requests for modified learning and assessment provisions. To aid understanding by all, use a variety of formats of communication (e.g. diagram vs text, paper vs online, verbal vs read). Where necessary, make adjustments to the format of communication for students with specific needs. Also, it may be appropriate to adjust the time that students have to either understand or express communications ( Ice et al., 2007). Adjustments should also be considered to accommodate for demographic differences ( Burr & Leung, 2015). An example of this would be to take account of different cultural expectations with respect to a didactic approaches to learning, and the acceptability of challenging a respected teacher. Many non-western cultures would emphasise respect to elders and teachers in a way that westerners do not. All of these communication issues need to be considered as all types of staff, not just teachers, interact with students. When considering student needs, it is important to take account of the power dynamic between student and teacher (Botas, 2011).

## Produce an annual timetable and tutorial touchstones to support continuous engagement

It is necessary to provide a structural scaffold for the exchange of information, ensuring it is of consistent quality and quantity, and spread evenly so that the workload is manageable, timely and usable ( Burr & Brodier, 2010). Regular tutorials need to be provided with clear agendas to ensure consistent coverage of the issues relevant at the stage of study. The tutorial can be a place for the dialogic feedback described by Nicol (2010) to occur. Thus, students should go through formative and summative information, with a teacher, to check that they understand what is being said to them and that they have planned how to respond to what has been said to them. The literature suggests that people often do not understand feedback in the same way as the person who produced it ( Pokomy & Pickford, 2010; Adcroft, 2011). If discussion regarding the use of information to guide learning is a major aspect of tutorials, this misunderstanding can be reduced. Combining performance information with reviews of learning analytics on engagement and with resources, can further help direct a more effective learning strategy ( Burr et al, 2013).

## Show students that their communications make a difference

Student participation in the decision making within the school can help in the development of what we do. We need student input and students need to know we need their input. It is important to understand student needs, and how students perceive what the school currently provides, when we decide any changes. It is equally important to help students understand the importance of their contribution so that they are encouraged to invest further in our joint endeavour. For us to value their ideas and opinions fosters their sense of belonging and shared ownership in the school. This in turn encourages continued engagement and ensures that the efforts of both staff and students is harnessed for the betterment of all. The student voice can be heard in both the discussion that students have with staff, and in pedagogic research. For example, the results from pedagogical research are often not shared with students, despite the fact that they may be key participants in the research process. Sharing the results of such research can empower students and encourage future participation in the activities of the school. It is thus helpful to show students that they can make a difference to the way that their course is delivered and show students how we have changed our activities in response to their feedback.

## Take Home Messages

The overall purpose of guided improvement in performance requires the provision of opportunities to learn, take corrective action, and try again. We believe this idea applies equally to the continuous learning of both the teachers and students within a medical school. The advice we have offered refer to a planned exchange of ideas based on mutual respect and openness. Sensitive teachers will always have listened to their students, modern practices are making this reality more explicit. What we suggest reflects good practice that may have been existent in places for years, even within didactic education, but would benefit from wider appreciation.

## Notes On Contributors

Tim Ley, is a Lecturer in Clinical Education (Psychology) at Peninsula Medical School, University of Plymouth.

Jolanta Kisielewska, PhD, is a Lecturer in Physiology at Peninsula Medical School, University of Plymouth.

Tracey Collett, PhD, is an Associate Professor (Education) at Peninsula Medical School, University of Plymouth.

Steven Ashley Burr, PhD, is an Associate Professor in Physiology and Deputy Director of Assessment at Peninsula Medical School, University of Plymouth.

## Declarations

The author has declared that there are no conflicts of interest.

## Ethics Statement

Not applicable as no data or individual information was involved.

## External Funding

This article has not had any External Funding
