# Supplementary material for: Improving communication for learning with students: expectations, feedback and feedforward
Source: MedEdPublish (2016). 2019 Jan 15;8:14. [Version 1] doi: 10.15694/mep.2019.000014.1 (PMC10712501; doi:10.15694/mep.2019.000014.1)
Supplement: Peer review history

# Reviewer report: Reviewer response for version 1

**DOI:** 10.21956/mep.19756.r29651

**Published:** 2019-06-23

**Authors:** Ken Masters

**Affiliations:** 1 Sultan Qaboos University

This review has been migrated. The reviewer awarded 4 stars out of 5 An interesting paper giving some basic tips on improving communication for learning with students. On the positive side, the paper does not confuse the reader with detailed theory, and this makes for easier reading. On the other hand, however, this does result in less grounding in the literature than one would like. This can sometimes result in statements that appear to be generalisations with little to support them. For example, “this is in marked contrast to the current norm where elements of the course are modularised into discrete units with separate informational processes.” Statements such these are not necessarily universally applicable, and should not be presented as such.One other small point of concern on the general layout: Frequently, the information is presented in large chunks of text, and the authors should consider breaking the wall into smaller paragraphs. This is important not only from an aesthetic point of view, but also to follow the argument, as each tip frequently involves several points that need to be individually digested, otherwise one seems to run into the next.Overall, however, a useful paper.

Reviewer Expertise:

NA

No decision status is available

# Reviewer report: Reviewer response for version 1

**DOI:** 10.21956/mep.19756.r29652

**Published:** 2019-01-28

**Authors:** Yukihiro Ikeda

**Affiliations:** 1 yuyu@med.kindai.ac.jp

This review has been migrated. The reviewer awarded 4 stars out of 5 Improving communication skills may be the most important issue for medical staff. This paper also focused on that point and was well inspected and written. I think that feedback to students is very important. Timing and appropriate words and phrases, how can students be able to improve? It was written in this article and it was helpful.

Reviewer Expertise:

NA

No decision status is available

# Reviewer report: Reviewer response for version 1

**DOI:** 10.21956/mep.19756.r29650

**Published:** 2019-01-15

**Authors:** Megan Anakin

**Affiliations:** 1 University of Otago

This review has been migrated. The reviewer awarded 4 stars out of 5 The title of this article caught my eye. I was pleasantly rewarded with a well-written and thoughtfully structured article. I found it to be informative and the authors offer well-reasoned suggestions. Being from Australasia, I missed seeing reference to the work of Liz Malloy and David Boud (For example see: Boud D. Feedback: Ensuring that it leads to enhanced learning. Clin Teach. 2015. 12(1), 3-7. Boud D. Molloy E. (Eds.). Feedback in higher and professional education: understanding it and doing it well. Routledge. 2013.) I am wondering if the authors are planning to design a study to investigate some of their suggested principles. I would be interested in the approaches they would take to implement and evaluate some of the ideas expressed in this article.

Reviewer Expertise:

NA

No decision status is available
